# Cyclic Octamer Peptoids: Simplified Isosters of Bioactive Fungal Cyclodepsipeptides

**DOI:** 10.3390/molecules23071779

**Published:** 2018-07-19

**Authors:** Assunta D’Amato, Giorgio Della Sala, Irene Izzo, Chiara Costabile, Yuichi Masuda, Francesco De Riccardis

**Affiliations:** 1 Department of Chemistry and Biology “A. Zambelli”, University of Salerno, Via Giovanni Paolo II, 132, 84084 Fisciano, Italy; asdamato@unisa.it (A.D.); gdsala@unisa.it (G.D.S.); iizzo@unisa.it (I.I.); ccostabile@unisa.it (C.C.); 2 Graduate School of Bioresources, Mie University, 1577 Kurimamachiya-cho, Tsu 514-8507, Japan

**Keywords:** cyclic peptoids, cyclodepsipeptides, insecticidal activity, conformational isomerism, structure-based design, peptidomimetics

## Abstract

Cyclic peptoids have recently emerged as an important class of bioactive scaffolds with unique conformational properties and excellent metabolic stabilities. In this paper, we describe the design and synthesis of novel cyclic octamer peptoids as simplified isosters of mycotoxin depsipeptides bassianolide, verticilide A1, PF1022A and PF1022B. We also examine their complexing abilities in the presence of sodium tetrakis[3,5-bis(trifluoromethyl)phenyl]borate (TFPB) salt and explore their general insecticidal activity. Finally, we discuss the possible relationship between structural features of free and Na^+^-complexed cyclic octamer peptoids and bioactivities in light of conformational isomerism, a crucial factor affecting cyclic peptoids’ biomimetic potentials.

## 1. Introduction

While the vast inventory of natural products finds its limits in the finiteness of biosynthetic pathways [1], the synthesis of non-natural analogues has no boundaries [2]. For their distinct conformational properties [3], their excellent biostabilities [4] and straightforward modular construction [5], peptoids (i.e., oligomers of *N*-substituted glycines) [6] represent a formidable class of biomimetic compounds with striking bio- and pharmacological activities [7,8].

Among peptoid oligomers, cyclic peptoids hold a special status for their intrinsic ability to adopt compact folded conformations [9,10] and exhibit conspicuous therapeutic properties [9,11]. Their excellent biomimetic properties have been confirmed by a recent study in which abiotic *hexamer* cyclopeptoids (mimicking depsipeptide mycotoxins of the enniatins class) evoked potent cytotoxic activities against cancer cell lines [12].

**Figure 1:**
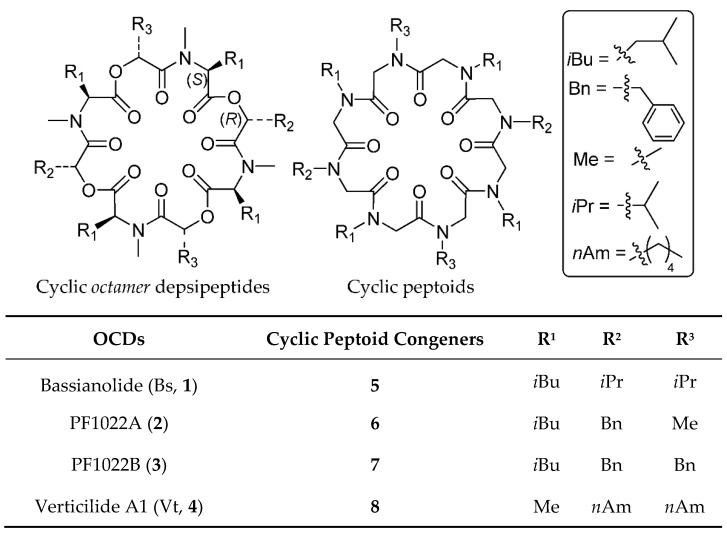
Natural octamer cyclic depsipeptides (OCDs) mycotoxins (**1**–**4**) and cyclic peptoid congeners (**5**–**8**) synthesized in the present paper.

In the present contribution, we report the design and synthesis of structural congeners of cyclic *octamer* depsipeptides bassianolide (Bs, **1**) [13,14], verticilide A1 (VtA1, **2**) [15,16] and PF1022A/PF1022B (**3** and **4**, respectively, Figure 1) [17,18,19,20]. In this investigation, we will evaluate both the advantages and drawbacks related to the use of cyclic peptoids as natural products mimics.

Peptoid congeners **5**–**8** (Figure 1) were obtained replacing the α-amino/hydroxy acids methines and intraannular N/O atoms present in natural cyclodepsipepides with nitrogen atoms and methylene groups, respectively. *N*-methyl substituents of l-leucine/alanine residues were removed in order to have a sequence of *N*-alkyl glycines. The relative position of the carbonyl groups was left unaltered to preserve possible metal chelating properties, essential for biological activities [14,20,21,22,23].

The depsipeptide/peptoid core switch yields congeners fully compatible with the solid-phase synthesis. Moreover, starting materials are inexpensive and the availability of hundreds of primary amines allows extensive structure-activity investigations.

On simplified isosters **5**–**8** general insecticidal assays on 4th-instar silkworm larvae [24,25] have been evaluated.

## 2. Results and Discussion

### 2.1. Chemistry and Conformational Studies

The syntheses of cyclic octamer peptoids **5**–**8** began with the solid-phase “submonomer” construction of linear precursors **9**–**12** (Table 1) [5]. *N*,*N*’-diisopropylcarbodiimide-induced bromoacetic acid condensations were alternated with amine substitutions until formation of the desired linear oligomeric amides. Detachment from the acid-labile solid support in the presence of hexafluoroisopropanol yielded octamers in acceptable to good yields and decent purities (Table 1).

Head-to-tail cyclisations of crude linear peptoids **9**–**12** were performed in high dilution conditions (3.0 mM concentration) in the presence of HATU as the coupling agent [26]. Cyclic compounds were purified from traces of unreacted/oligomerized linear peptoids through precipitation from hot acetonitrile (in the case of **5** and **7**) or reverse-phase column chromatography (oligomers **6** and **8**). Cyclic oligoamides **5**–**8** were isolated as white amorphous solids (purity > 98%, HPLC analysis).

**Figure 2:**
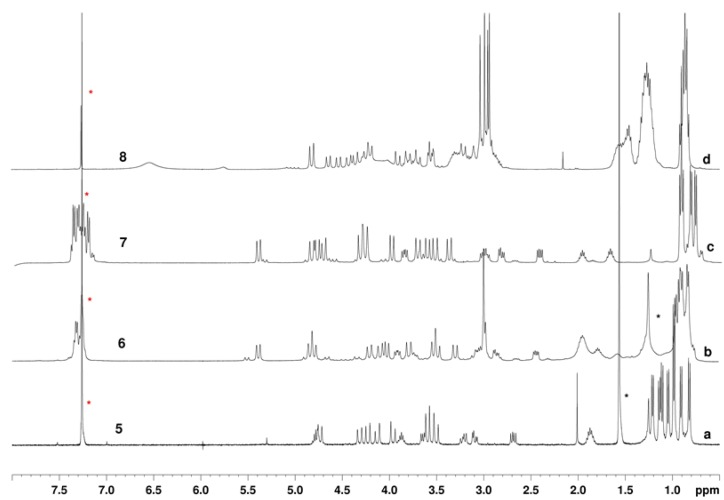
^1^H NMR spectra of cyclic peptoids **5**–**8**. 5.0–10.0 mM solutions in CDCl_3_ (400 MHz). Residual solvent peaks are labelled with a red asterisk. H_2_O and further CDCl_3_ impurities are labelled with a black asterisk.

Despite their large size, the mycotoxins’ congeners showed good conformational stabilities (^1^H NMR analysis, Figure 2). In particular, cyclopeptoids with β-branched *N*-side chains (**5**–**7**) exhibited single dominant conformers (>85%, ^1^H NMR analysis, Figure 2a–c), while *N*-methyl/amyl substituted **8** displayed multiple conformations in slow equilibrium on the NMR time scale (Figure 2d).

Conformational interconversion barriers have been quantified by variable-temperature (VT) ^1^H NMR experiments for conformationally homogeneous **5**–**7**. Coalescence temperatures were experimentally determined to be 110 °C (C_2_D_2_Cl_4_, ΔG^‡^ = 17.4 ± 0.9 kcal/mol, 300 MHz), 90 °C (C_2_D_2_Cl_4_, ΔG^‡^ = 15.9 ± 0.8 kcal/mol, 300 MHz) and 90 °C (C_2_D_2_Cl_4_, ΔG^‡^ = 16.4 ± 0.8 kcal/mol, 300 MHz) for cyclopeptoids **5**, **6** and **7**, respectively. No conformational stability studies have been reported for octamer cyclodepsipeptides in that, in most of the deuterated solvents, bassianolide [14], PF1022A [15,27] and verticilide A1 [28] are present as complex mixtures of conformers in slow equilibrium on the NMR time scale.

**Figure 3:**
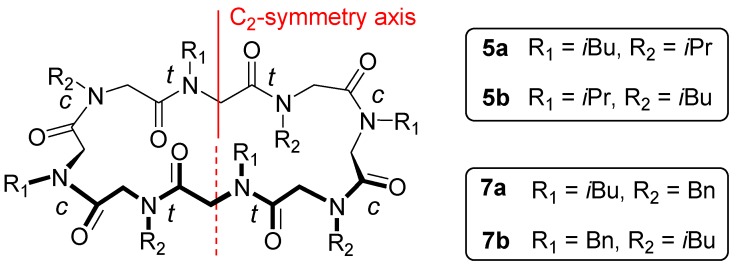
Schematic structure of the possible conformational diastereoisomers for cyclic peptoids **5** and **7**.

2D homonuclear (COSY) and heteronuclear (HMQC, HMBC) experiments allowed assignment of the ^1^H/^13^C resonances for cyclic peptoids **5**–**7**, suggesting formation of *C*_2_-symmetric *ccttcctt* species for **5** and **7** (half of the signals were present in the NMR spectra, as previously reported for octamer peptoids [29,30,31,32,33,34]) and a *C*_1_-symmetric species for **6**. Peptoid bond geometries have been documented by ^1^H NMR chemical shifts values. In particular, relatively low values for Cα-H *N*-side chains’ or small Δδ for diastereotopic Cα-H_2_ testified for *trans* peptoid junctions; higher values of chemical shifts or larger Δδ were indicative of *cis* amide bonds [12]. The NMR data identified two possible alternative structures for symmetric **5** and **7**. In these congeners two of the four isobutyl/isopropyl and benzyl side chains were located on *cis* amide bonds and the other two were on *trans* peptoid junctions (**5a**/**5b** and **7a**/**7b**, Figure 3, see also Figure S2 of Supplementary Material).

Accurate analysis of the ^1^H NMR resonances showed no symmetry elements for compound **6** (to the best of our knowledge, this is the first case of symmetry breaking in the case of a conformationally stable, symmetrically substituted, cyclic octamer peptoid) (see experimental part and Figure S2 of Supplementary Material). The presence of two *N*-benzyl and two *N*-isobutyl side chains on *cis* amide bonds, plus two *N*-isobutyl and two *N*-methyl side chains on *trans* peptoid junctions testified for the asymmetric arrangement of cyclic oligomer **6**.

**Figure 4:**
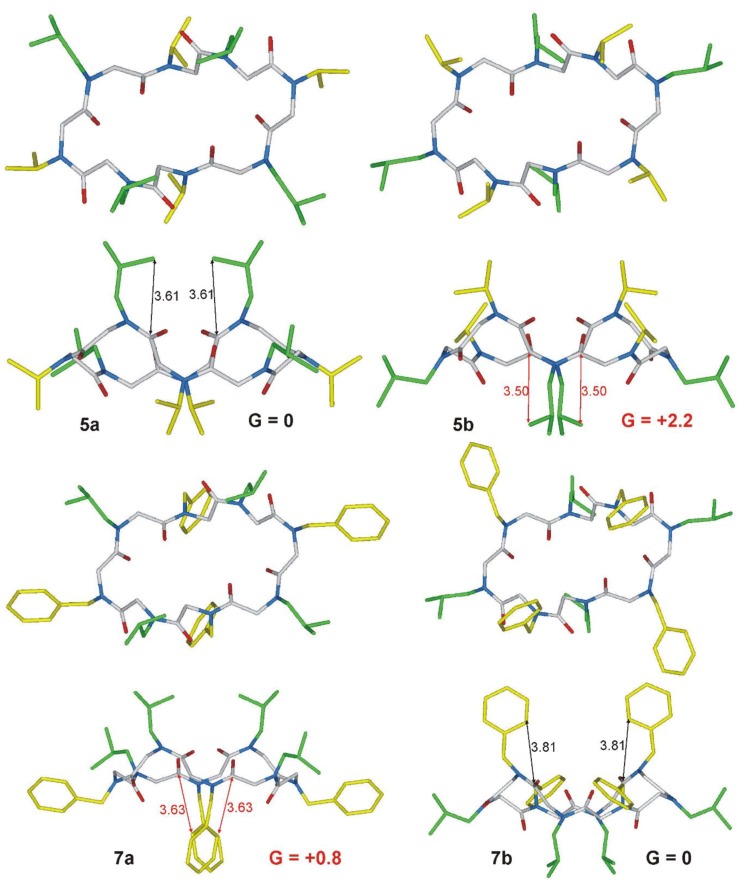
Top and side view of minimum energy structures of conformational diastereisomers **5a/b** and **7a/b**. For the sake of clarity isobutyl groups are reported in green, whereas isopropyl and benzyl groups in yellow. Free energies were calculated in CHCl_3_ and reported in kcal/mol. Distances are reported in Å.

*C*_2_-symmetric *ccttcctt*-conformational diastereoisomers **5a/b** and **7a/b** were modelled by DFT (Density Functional Theory) studies and minimum energy structures were located in order to find the plausible most stable conformations. Free energies in CHCl_3_ have been reported in Figure 4 (see S.M. for computational details). Isomers **5a** and **7b** were found to be more stable than the corresponding conformational diastereoisomers **5b** and **7a** of 2.2 and 0.8 kcal/mol, respectively. The energy differences are mainly due to steric interactions among the *N*-substituents and the ring. More in detail, as reported in Figure 4, **5b** shows isobutyl-ring distances shorter than **5a**, whereas interactions among the phenyl groups and the ring is responsible for the higher energy of **7a** with respect to **7b**. Furthermore, the higher energy difference between **5a** and **5b** (2.2 kcal/mol) could explain why the NMR spectrum of **5** shows only one conformational isomer, whereas the ^1^H NMR spectrum of **7** shows a small amount of the other isomer (Figure 2c). These data are also concordant with the VT-experiments (the coalescent temperature and therefore the conformational interconversion barrier, is higher for cyclopeptoid **5**).

It is interesting to note that the removal of two phenyl groups in compounds **6** (where two *N*-methyl groups take the place of two *N*-benzyl side chains in **7**) causes a profound effect on the intrinsic stability of the cyclopeptoid framework and breaks the symmetry of the octameric species.

Another important aspect regarding the conformational isomerism of rigid cyclic octamer peptoids is related to their conformational chirality [10].

The presence of a *C*_1_- or *C*_2_-symmetry axis and the relatively high barrier for conformational inversion of the cyclooligomeric species (ΔG^‡^ ≥ 10 kcal/mol) makes cyclic peptoid **5**–**7** chiral and observable as conformational enantiomers in solution by NMR spectroscopy (which, at room temperature, reveals species with lifetimes exceeding 10^–2^ s) [10].

**Figure 5:**
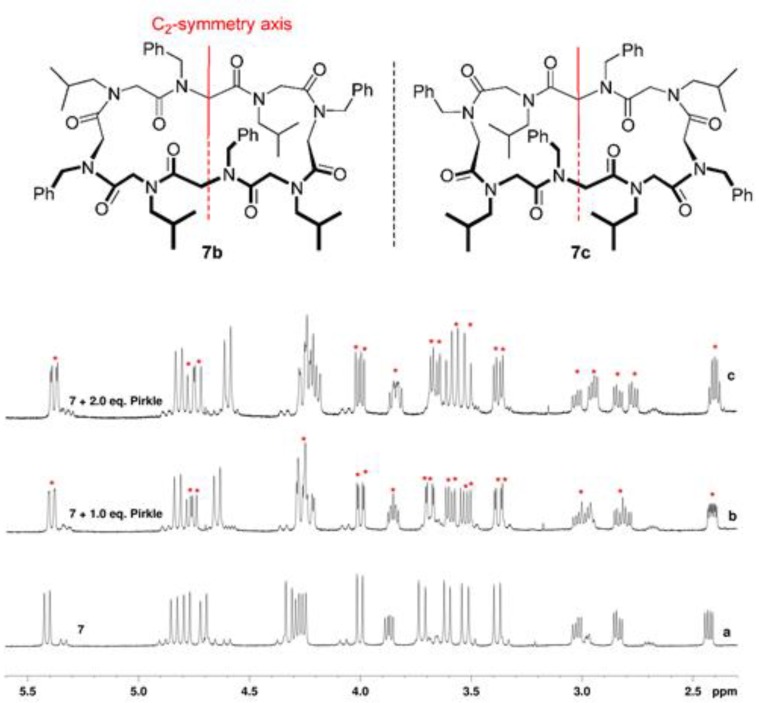
^1^H NMR spectra resulting from the quantitative step-wise addition of Pirkle’s alcohol to the 5.0 mM solution in CDCl_3_ (600 MHz). Red asterisks denote split signals.

The presence of enantiomorphic conformers was demonstrated by step-wise addition of Pirkle’s alcohol ((*R*)-1-(9-anthryl)-2,2,2-trifluoroethanol) [35] to a CDCl_3_ solution of representative cyclopeptoid **7** (Figure 5). Extensive signal splitting (marked with red asterisks) confirmed the presence in solution of both enantiomers **7b**/**7c**.

### 2.2. Complexation Studies

**Figure 6:**
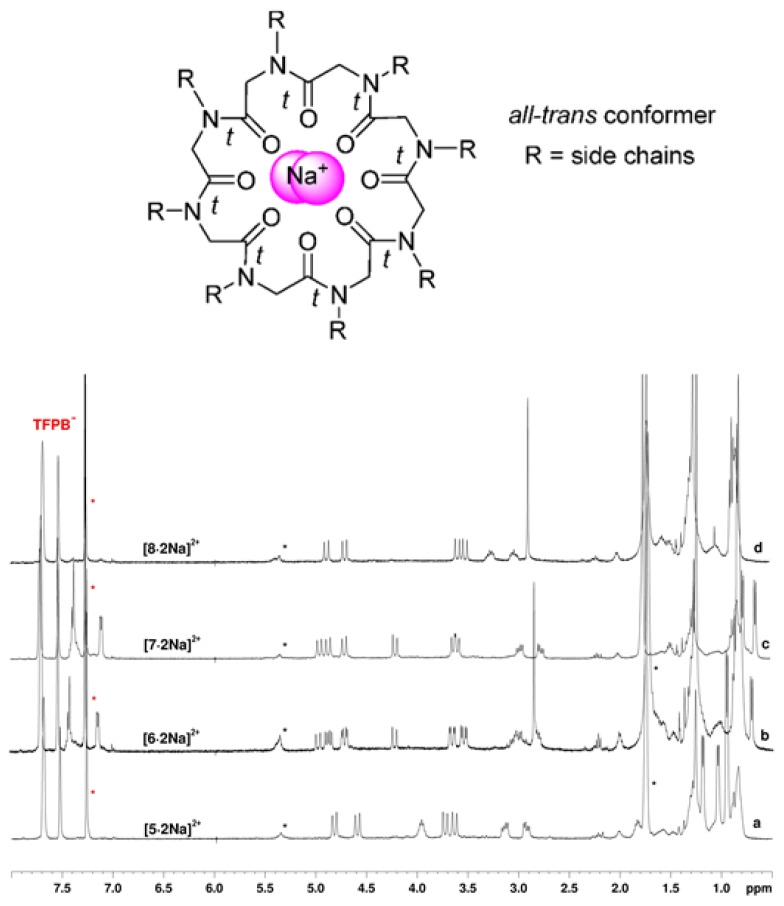
^1^H NMR spectra showing [**5**–**8** 2Na]^2+^ complexes and general structure of the highly symmetric *all-trans* core conformer. Residual solvent peaks are labelled with a red asterisk. H_2_O and further CDCl_3_ impurities are labelled with a black asterisk. 1.0 mM host solutions in the presence of 2.0 equivalents of NaTFPB in CDCl_3_ (400 MHz).

The biological action of CDPs is often associated to the formation of metal complexes [18,19,20]. We therefore tested the metal chelating attitudes of cyclic peptoids **5**–**8** in the presence of sodium tetrakis[3,5-bis(trifluoromethyl)phenyl]borate (NaTFPB, [12]) in CDCl_3_ solutions (^1^H NMR analysis, Figure 6). In all the performed experiments the presence of two equivalents of Na^+^ cation induced formation of highly symmetric metalated species in slow equilibrium with the corresponding free hosts on the NMR time scale. Addition of one equivalent of NaTFPB showed the formation of a mixture of multiple complexes in slow equilibrium on the NMR time scale. Two equivalent of cationic guest established the formation of single detectable, highly symmetric, complexes. The relatively low chemical shift values observed for the *N*Ala (2.85 ppm in [**6**·2Na]^2+^; 2.88 ppm in [**8**·2Na]^2+^) and *N*Val (3.96 ppm in [**5**·2Na]^2+^) CαH resonances; the small Δδ evidenced for the diastereotopic -N-C*H_2_*-CH(CH_3_)_2_ protons of *N*Leu (0.22 in [**5**·2Na]^2+^; 0.30 in [**6**·2Na]^2+^), -N-C*H_2_*-Ph protons of *N*Phe (0.48 in [**6**·2Na]^2+^; 0.51 in [**7**·2Na]^2+^) and -N-C*H_2_*-(CH_2_)_3_-CH_3_ protons of *N*am (0.23 in [**8**·2Na]^2+^) residues suggested, for all the cyclic octamers, *all-trans* peptoid junctions [36].

The host/guest ratio (calculated integrating the signals of the host/guest complex respect to those of dissolved guest, NaTFPB) remained constant during all the titration experiments and documented a 1:2 macrocycle/Na^+^ *ratio* for all the synthesized complexes. The apparent association constants were therefore calculated for the formation of the [**5**–**8**·2Na]^2+^ species by ^1^H NMR (1.0 mM, CDCl_3_) [37,38] showing similar values for all the bimetallic adducts (K_a_: [**5**·2Na]^2+^: 1.9·10^7^ M^−2^, [**6**·2Na]^2+^: 7.2·10^6^ M^−2^, [**7**·2Na]^2+^: 1.9·10^6^ M^−2^, [**8**·2Na]^2+^: 1.3·10^7^ M^−2^). VT ^1^H NMR experiments indicated no sign of coalescence up to 110 °C (C_2_D_2_Cl_4_, 600 MHz). The efficient ion-dipole interactions between the carbonyl groups and the sodium cation increases the conformational stabilities of the macrorings. 

**Figure 7:**
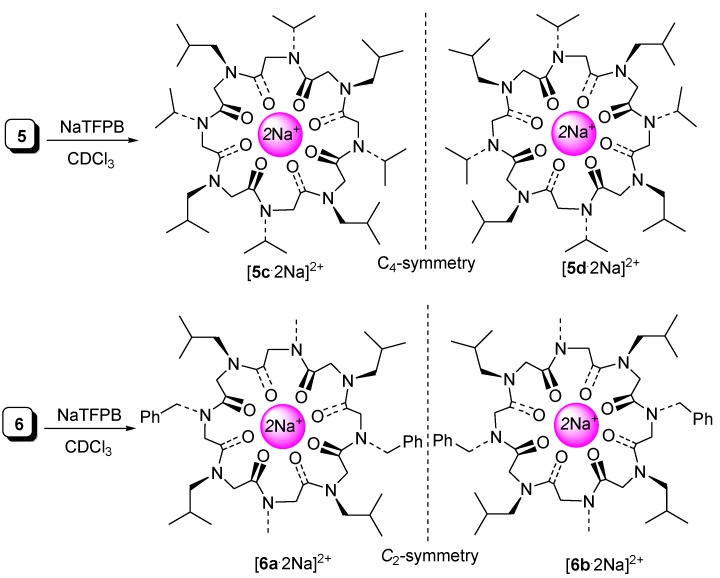
Schematic structures of both the enantiomers of *all*-*trans* Na^+^-complexes of cyclic octamer peptoids **5** and **6**. *C*_4_-symmetric [**5c·**2Na]^2+^/[**5d·**2Na]^2+^and *C*_2_-symmetric [**6a**·2Na]^2+^/[**6b**·2Na]^2+^ are non-superimposable mirror images. Amide bonds are considered as rigid planes.

Also, the Na^+^-complexes were present as couples of enantiomorphous cyclooligomers (Figure 7). In particular, cyclic peptoids [**5c**·2Na]^2+^ and [**5d**·2Na]^2+^, showing alternating side chains, are *C*_4_-symmetric conformational enantiomers; macrorings [**6a**·2Na]^2+^ and [**6b**·2Na]^2+^ are *C*_2_-symmetric species and **7** and **8** (not reported), with alternating side chains, form couples of *C*_4_-symmetric conformational enantiomers.

Formation of conformational enantiomers in rigid octamer cyclopeptoids as free hosts or complexes represents a crucial factor to predict/interpret their biological action. It should be noted that the presence of enantiomorphous species is not detrimental to the biological activity only when the biological target is not chiroselective (as in the case of the cell membrane) [39]. Things change dramatically when the target of biological action is a chirospecific receptor (proteins, carbohydrates or nucleic acids).

In the case of enniatin A and B, both enantiomers are bioactive [40] (cyclic hexadepsipeptides, in fact, interfere with the cations’ transport through the membrane). Studies on the molecular action of octamer depsipetides indicate specific molecular targets for these molecules. It is known for example, that PF1022A acts on the latrophin-like receptor and on the Ca^2+^-activated K^+^ channel [20]. Verticilide A1 has been shown to bind selectively to the insect ryanodine receptor, a major target for modern insecticides [15] and it efficiently inhibits the acyl-CoA cholesterol acyltransferases ACAT1 and ACAT2 [41]. Emodepside, a broad spectrum anthelmintic cyclooctadespispetide derived from PF1022A [21], has multiple molecular targets. On the basis of these considerations, it is clear that the presence of stable free and complexed conformationally stable diastereomers and enantiomers is detrimental for the potential bioactivity.

At this point, conscious of the intrinsic drawbacks due to the conformational and stereochemical dispersion of the synthesized cyclic peptoids, we decided to perform a test on silkworm larvae in order to evaluate general insecticidal activity.

### 2.3. Insecticidal Activity against Silkworm Larvae

Bassianolide (**1**) was reported to exhibit acute lethal toxicity against silkworm larvae [14]. We tested insecticidal activity of **5**, a structural congener of bassianolide and analogues **6**–**8**. Each peptoid in dimethyl sulfoxide (DMSO) was injected into open vessels of 4th-instar larvae. The administration of **5**–**8** caused death of a few larvae at a dose of 300 nmol/larva after 72–120 h (Table 2). Their toxicities were much weaker than that of bassianolide (**1**), which induced atonic symptom within 0.5 h and 100% mortality in a week at a dose of 5 μg (=5.5 nmol)/larva [14]. 

Anti−proliferative potentials of the **5**–**8** were tested on A375 (human melanoma) cancer cell line. The cells were incubated for 72 h with increasing concentration of compounds (10–25–50 µM) and cell viability was determined by MTT proliferation assay. The data indicated that the assayed compounds did not affect the cell vitality (see experimental part for general procedures).

## 3. Experimental Section

### 3.1. Chemistry

#### 3.1.1. General Methods

Starting materials and reagents purchased from commercial suppliers were generally used without purification unless otherwise mentioned. HPLC analyses were performed on a JASCO LC-NET II/ADC equipped with a JASCO Model PU-2089 Plus Pump and a JASCO MD-2010 Plus UV-vis multiple wavelength detector set at 220 nm. The column used was a C_18_ reversed-phase analytical column (Waters, Bondapak, 10 μm, 125 Å, 3.9 mm × 300 mm) run with linear gradients of ACN (0.1% TFA) into H_2_O (0.1% TFA) over 30 min, at a flow rate of 1.0 mL/min for the analytical runs. ESI-MS analysis in positive ion mode was performed using a Finnigan LCQ Deca ion trap mass spectrometer (ThermoFinnigan, San Josè, CA, USA) and the mass spectra were acquired and processed using the Xcalibur software provided by Thermo Finnigan. Samples were dissolved in 1:1 CH_3_OH/H_2_O, 0.1% formic acid and infused in the ESI source by using a syringe pump; the flow rate was 5 μL/min. The capillary voltage was set at 4.0 V, the spray voltage at 5 kV and the tube lens offset at −40 V. The capillary temperature was 220 °C. Data were acquired in MS1 and MSn scanning modes. Zoom scan was used in these experiments. High-resolution mass spectra (HRMS) were recorded on a Bruker Solarix XR Fourier transform ion cyclotron resonance mass spectrometer (FTICR-MS) equipped with a 7T magnet, using electrospray ionization (ESI). Yields refer to chromatographically and spectroscopically (^1^H- and ^13^C NMR) pure materials. NMR spectra were recorded on a Bruker DRX 600 (^1^H at 600.13 MHz, ^13^C at 150.90 MHz), Bruker DRX 400 (^1^H at 400.13 MHz, ^13^C at 100.03 MHz), Bruker DRX 300 (^1^H at 300.13 MHz, ^13^C at 75.03 MHz). Chemical shifts (δ) are reported in ppm relative to the residual solvent peak (CHCl_3_, δ = 7.26; ^13^CDCl_3_, δ = 77.00; C_2_DHCl_4_, TCDE, δ = 5.80) and the multiplicity of each signal is designated by the following abbreviations: s, singlet; d, doublet; dd, double doublet; t, triplet; sept, septet; m, multiplet; br, broad. 2D NMR experiments such as COSY, ROESY, HSQC and HMBC were performed for the full assignment of each signal. Coupling constants (*J*) are quoted in Hertz.

#### 3.1.2. General Procedure for the Solid-Phase Synthesis of Linear Peptoids **9**–**12**

The 2-chlorotrityl chloride resin (α-dichlorobenzhydryl-polystyrene cross-linked with 1% DVB; 100–200 mesh; 1.63 mmol g^–1^, 0.400 g, 0.652 mmol) was swelled in dry CH_2_Cl_2_ (4 mL) for 45 min and washed twice with dry CH_2_Cl_2_ (4 mL). The first submonomer was attached onto the resin by adding bromoacetic acid (0.136 g, 0.978 mmol) in dry CH_2_Cl_2_ (4 mL) and DIPEA (567 μL, 3.26 mmol) on a shaker platform for 60 min at room temperature, followed by washing with CH_2_Cl_2_ (3 × 1 min) and then again with DMF (3 × 1 min). A solution of the chosen amine (1.6 M in dry DMF, 4 mL) was added to the bromoacetylated resin. The mixture was left on a shaker platform for 40 min at room temperature, then the resin was washed with DMF (3 × 1 min), CH_2_Cl_2_ (3 × 1 min) and then again with DMF (3 × 1 min). Subsequent bromoacetylation reactions were accomplished by reacting the aminated oligomer with a solution of bromoacetic acid (0.910 g, 6.52 mmol) and DIC (1.11 mL, 7.17 mmol) in dry DMF (4 mL) for 60 min at room temperature. The completion of the acylation reactions was verified by using the chloranil test. The filtrated resin was washed with DMF (3 × 1 min), CH_2_Cl_2_ (3 × 1 min), DMF (3 × 1 min) and treated again with the proper amine under the same conditions reported above. This cycle of reactions was iterated until the target linear oligomer was obtained. The cleavage was performed treating the resin, previously washed with CH_2_Cl_2_ (3 × 1 min), three times with a solution of HFIP in CH_2_Cl_2_ (20% *v*/*v*, 4.0 mL each time) on a shaker platform at room temperature for 30 min each time. The resin was then filtered away and the combined filtrates were concentrated in vacuo. 1 mg of the final products were dissolved in 60 μL of acetonitrile (0.1% TFA) and 60 μL of HPLC grade water (0.1% TFA) and analysed by RP-HPLC; purity ≥ 71%; conditions: 5 → 100% A in 30 min for the all oligomers (A, 0.1% TFA in acetonitrile, B, 0.1% TFA in water); flow: 1.0 mL min^−1^, 220 nm]. The linear oligomers (isolated as amorphous solids) were subjected to ESI mass spectrometry (see Table 1) and, subsequently, to the cyclization reactions without further purification.

#### 3.1.3. General Procedure for the High Dilution Cyclization. Synthesis of Cyclic Peptoids **5**–**8**

The solutions of the linear peptoids (0.150 mmol), previously co-evaporated three times with toluene, were prepared under nitrogen in dry DCM (5.0 mL). The mixture was added dropwise to a stirred solution of HATU (0.171 g, 0.450 mmol) and DIPEA (104 μL, 0.600 mmol) in dry DMF (45.0 mL) by a syringe pump in 5 h, at room temperature in anhydrous atmosphere. After 12 h the resulting mixture was concentrated *in vacuo*, diluted with CH_2_Cl_2_ (30 mL) and washed with a solution of HCl (1.0 M, 15 mL). The aqueous phase was then extracted again with 30 mL of CH_2_Cl_2_. The organic phases were washed with water (60.0 mL), dried over anhydrous MgSO_4_, filtered and concentrated *in vacuo*. The cyclic peptoids were dissolved in 50% acetonitrile in HPLC grade water and analysed by RP-HPLC; purity > 90% conditions: 5–100% A in 30 min (A, 0.1% TFA in acetonitrile, B, 0.1% TFA in water); flow: 1 mL min^−1^, 220 nm. The crude cyclic peptoids **5**, **7** were dissolved in hot acetonitrile and precipitated by slowly cooling the acetonitrile solutions. The crude **6**, **8** were purified on reverse silica gel (C_18_); conditions: 10–100% A (A: acetonitrile; B: water).

**5**: white amorphous solid, 0.027 g, yield: 21%; *t_R_*: 12.1 min. ^1^H-NMR (400 MHz, CDCl_3_, *c* stands for *cis* amide bond, *t* stands for *trans* amide bond) δ: 4.77 (2H, m, N-C*H*(CH_3_)_2_, *c*), 4.74 (2H, d, *J* 16.1 Hz, O=C-C*H*H-N-*i*Bu, *t*), 4.32 (2H, d, *J* 17.8 Hz, O=C-C*H*H-N-*i*Bu, *c*), 4.23 (2H, d, *J* 17.7 Hz, O=C-C*H*H-N-*i*Pr, *t*), 4.13 (2H, d, *J* 17.8 Hz, O=C-C*H*H-N-*i*Pr, *c*), 3.96 (2H, d, *J* 17.8 Hz, O=C-CH*H*-N-*i*Pr, *c*), 3.87 (m, 2H, N-C*H*(CH_3_)_2_, *t*), 3.64 (2H, dd, *J* 14.1, 7.4 Hz, N-C*H*H-CH(CH_3_)_2_, *c*), 3.60 (4H, d, O=C-CH*H*-N-*i*Bu, *t* and O=C-CH*H*-N-*i*Bu, *c*, overlapping), 3.51 (2H, d, *J* 17.7 Hz, O=C-CH*H*-N-*i*Pr, *t*), 3.22 (2H, dd, *J* 13.9, 5.0 Hz, N-C*H*H-CH(CH_3_)_2_, *t*), 3.09 (2H, dd, *J* 13.9, 5.0 Hz, N-CH*H*-CH(CH_3_)_2_, *t*), 2.68 (2H, dd, *J* 14.1, 7.4 Hz, N-CH*H*-CH(CH_3_)_2_, *c*), 1.87 (4H, m, N-CH_2_-C*H*(CH_3_)_2_, *c* and *t*), 1.21 (6H, d, *J* 6.7 Hz, N-CH(C*H_3_*)*_2_*, *t*), 1.14 (6H, d, *J* 6.7 Hz, N-CH(C*H_3_*)*_2_*, *c*), 1.11 (6H, d, *J* 6.7 Hz, N-CH(C*H_3_*)*_2_*, *t*), 1.05 (6H, d, *J* 6.7 Hz, N-CH(C*H_3_*)*_2_*, *c*), 0.98 (12H, d, *J* 6.5 Hz, N-CH_2_-CH(C*H_3_*)*_2_*, *c* and *t*), 0.91 (6H, d, *J* 6.5 Hz, N-CH_2_-CH(C*H_3_*)*_2_*, *t*), 0.82 (6H, d, *J* 6.5 Hz, N-CH_2_-CH(C*H_3_*)*_2_*, *c*). ^13^C-NMR (100 MHz, CDCl_3_) δ: 170.3 (x2), 169.4 (x2), 169.3 (x2), 166.1 (x2), 56.2 (x2), 55.8 (x2), 50.6 (x2), 49.0 (x2), 48.0 (x2), 45.6 (x2), 43.2 (x2), 42.8 (x2), 27.8 (x2), 27.0 (x2), 20.9 (x2), 20.6 (x3), 20.4 (x3), 19.9 (x4), 19.6 (x2), 19.6 (x2). HR-ESI-MS *m*/*z*: 849.6170 [M + H]^+^ (calcd. for C_44_H_81_N_8_O_8_^+^, 849.6172).

**6**: white amorphous solid, 0.041 g, yield: 31%; *t_R_*: 12.6 min. ^1^H-NMR (400 MHz, CDCl_3_, *c* stands for *cis* amide bond, *t* stands for *trans* amide bond) δ: 7.36-7.34-7.23 (10H, overlapping, Ar-*H)*, 5.52 (1H, d, *J* 15.6 Hz, N-C*H*H-Ph, *c*), 5.39 (1H, d, *J* 14.5 Hz, N-C*H*H-Ph, *c*), 4.91 (1H, d, *J* 16.8 Hz, O=C-C*H*H-N-CH_3_, *t*), 4.84 (1H, d, *J* 17.0 Hz, O=C-C*H*H-N-CH_3_, *t*), 4.71 (1H, d, *J* 17.8 Hz, O=C-C*H*H-N-*i*Bu, *t*), 4.67 (1H, d, *J* 16.0 Hz, O=C-C*H*H-N-*i*Bu, *t*), 4.37 (1H, d, *J* 16.8 Hz, O=C-C*H*H-N-*i*Bu, *c*), 4.22 (1H, d, *J* 17.9 Hz, O=C-C*H*H-N-Bn, *c*), 4.14 (1H, d, *J* 15.6 Hz, O=C-C*H*H-N-Bn, *c*), 4.09 (1H, d, *J* 18.3 Hz, O=C-C*H*H-N-*i*Bu, *c*), 4.03 (1H, d, *J* 14.5 Hz, N-CH*H*-Ph, *c*), 3.92 (1H, dd, *J* 13.5, 7.2 Hz, N-C*H*H-CH(CH_3_)_2_, *c*), 3.79 (1H, d, *J* 18.5 Hz, O=C-CH*H*-N-*i*Bu, *c*), 3.77 (2H, d, *J* 15.6 Hz, N-CH*H*-Ph, *c* and O=C-C*H*H-N-Bn, *c*, overlapping), 3.75 (1H, m, overlapping signals, N-C*H*H-CH(CH_3_)_2_, *c*), 3.71 (1H, d, *J* 16.8 Hz, O=C-CH*H*-N-*i*Bu, *c*), 3.52 (1H, d, *J* 16.0 Hz, O=C-CH*H*-N-*i*Bu, *t*), 3.51 (1H, d, *J* 17.0 Hz, O=C-CH*H*-N-CH_3_, *t*), 3.48 (1H, d, *J* 17.9 Hz, O=C-CH*H*-N-Bn, *c*), 3.30 (1H, d, *J* 17.8 Hz, O=C-CH*H*-N-*i*Bu, *t*), 3.10 (1H, d, *J* 16.8 Hz, O=C-CH*H*-N-CH_3_, *t*), 3.05 (1H, dd, *J* 13.2, 6.5 Hz, N-C*H*H-CH(CH_3_)_2_, *t*), 3.00 (3H, s, N-CH_3_, *t*), 2.99 (2H, m, overlapping signals, N-C*H_2_*-CH(CH_3_)_2_, *t*), 2.97 (3H, s, N-C*H_3_*, *t*), 2.96 (1H, dd, *J* 13.2, 6.5 Hz, N-CH*H*-CH(CH_3_)_2_, *t*), 2.87 (1H, dd, *J* 14.0, 7.6 Hz, N-CH*H*-CH(CH_3_)_2_, *c*), 2.44 (1H, dd, *J* 13.5, 7.2 Hz, N-CH*H*-CH(CH_3_)_2_, *c*), 1.97-1.72 (4H, m, overlapping signals, N-CH_2_-C*H*(CH_3_)_2_, *c* and *t*), 0.97-0.77 (24H, overlapping signals, N-CH_2_-CH(C*H_3_*)*_2_*, *c* and *t*). ^13^C-NMR (150 MHz, CDCl_3_) δ: 170.2, 169.8, 169.6, 169.3 (x2), 168.5, 167.2, 166.7, 136.6 (x2), 129.2, 129.0 (x3), 128.8 (x2), 128.6, 127.8, 127.4, 127.0, 56.1 (x2), 55.8, 55.1, 52.0, 50.9, 50.8, 50.4, 50.1, 49.8, 49.5, 49.2, 48.0, 46.9, 36.8, 35.4, 29.7, 27.8, 27.2 (x2), 20.6 (x2), 20.5, 20.3 (x2), 20.0, 19.9, 19.7. HR-ESI-MS *m*/*z*: 889.5543 [M + H]^+^ (calcd. for C_48_H_73_N_8_O_8_^+^, 889.5546).

**7**: white amorphous solid, 0.045 g, yield: 29%; *t_R_*: 14.6 min. ^1^H-NMR (400 MHz, CDCl_3_, *c* stands for *cis* amide bond, *t* stands for *trans* amide bond) δ: 7.36-7.18 (20H, overlapping, Ar-*H)*, 5.40 (2H, d, *J* 15.0 Hz, N-C*H*H-Ph, *c*), 4.83 (2H, d, *J* 17.3 Hz, O=C-C*H*H-N-*i*Bu, *t*), 4.77 (2H, d, *J* 16.1 Hz, O=C-C*H*H-N-Bn, *t*), 4.71 (2H, d, *J* 16.6 Hz, N-C*H*H-Ph, *t*), 4.32 (2H, d, *J* 17.8 Hz, N-CH*H*-Ph, *t*), 4.27 (4H, d, *J* 19.0 Hz, O=C-C*H*H-N-Bn, *c* and O=C-C*H*H-N-*i*Bu, *c*, overlapping), 3.98 (2H, d, *J* 15.0 Hz, N-CH*H*-Ph, *c*), 3.85 (2H, dd, *J* 13.6, 7.2 Hz, N-C*H*H-CH(CH_3_)_2_, *c*), 3.71 (2H, d, *J* 19.0 Hz, O=C-CH*H*-N-*i*Bu, *c*), 3.61 (2H, d, *J* 16.1 Hz, O=C-CH*H*-N-Bn, *t*), 3.53 (2H, d, *J* 19.0 Hz, O=C-CH*H*-N-Bn, *c*), 3.38 (2H, d, *J* 17.3 Hz, O=C-CH*H*-N-*i*Bu, *t*), 3.03 (2H, dd, *J* 14.9, 7.6 Hz, N-C*H*H-CH(CH_3_)_2_, *t*), 2.83 (2H, dd, *J* 14.9, 7.6 Hz, N-CH*H*-CH(CH_3_)_2_, *t*), 2.42 (2H, dd, *J* 13.6, 7.2 Hz, N-CH*H*-CH(CH_3_)_2_, *c*), 1.97 (2H, m, N-CH_2_-C*H*(CH_3_)_2_, *t*), 1.68 (2H, m, N-CH_2_-C*H*(CH_3_)_2_, *c*), 0.92 (12H, t, *J* 7.3 Hz, N-CH_2_-CH(C*H_3_*)*_2_*, *t*, overlapping signals), 0.82 (6H, d, *J* 6.5 Hz, N-CH_2_-CH(C*H_3_*)*_2_*, *c*), 0.77 (6H, d, *J* 6.5 Hz, N-CH_2_-CH(C*H_3_*)*_2_*, *c*). ^13^C-NMR (100 MHz, CDCl_3_) δ: 169.8 (x2), 169.7 (x2), 169.3 (x2), 167.4 (x2), 136.6 (x2), 135.9 (x2), 129.1 (x4), 128.8 (x4), 128.6 (x4), 128.0 (x2), 127.3 (x2), 126.7 (x4), 55.8 (x2), 55.1 (x2), 52.3 (x2), 50.1 (x2), 49.7 (x2), 49.4 (x2), 48.0 (x2), 47.1 (x2), 27.2 (x2), 20.4 (x2), 20.3 (x2), 20.2 (x2), 19.9 (x4). HR-ESI-MS *m*/*z*: 1041.6171 [M + H]^+^ (calcd. for C_60_H_81_N_8_O_8_^+^, 1041.6172).

**8**: white amorphous solid, 0.040 g, yield: 34%; *t_R_*: 13.3 min. ^1^H-NMR (400 MHz, CDCl_3_, mixture of rotamers) δ: 4.84-2.94 (36H, overlapping signals, O=C-C*H_2_*-N-CH_3_, O=C-C*H*_2_-N-CH_2_CH_2_CH_2_CH_2_CH_3_, N-C*H_2_*CH_2_CH_2_CH_2_CH_3_, O=C-CH_2_-N-C*H*_3_), 1.58–1.44 (8H, br signals, overlapping, N-CH_2_C*H_2_*CH_2_CH_2_CH_3_), 1.33–1.22 (16H, overlapping signals, N-CH_2_CH_2_C*H_2_*C*H_2_*CH_3_), 0.92–0.83 (12H, overlapping signals, N-CH_2_CH_2_CH_2_CH_2_C*H_3_*); ^13^C-NMR (100 MHz, CDCl_3_) δ: 169.9, 169.3, 169.2, 169.0, 168.0, 167.0, 51.6, 50.8, 50.5, 50.3, 49.0, 48.9, 48.6, 48.4, 48.0, 47.7, 36.6, 35.9, 35.5, 29.1, 29.0, 28.9, 28.7, 28.5, 27.5, 27.3, 27.0, 22.4, 22.2, 14.0. HR-ESI-MS *m*/*z*: 793.5540 [M + H]^+^ (calcd. for C_40_H_73_N_8_O_8_^+^, 793.5546).

#### 3.1.4. General Procedure for the Na^+^ Complexes Formation [5–8·2Na]^2+^·2[TFPB]^2^¯

To a solution of cyclic peptoids **5**–**8** in CDCl_3_ (0.9 mL) 2.0 equivalents of NaTFPB (previously dissolved in CD_3_CN, 0.1 mL) were added. After the addition, the mixtures were sonicated for 5 min in a heated bath (25 °C). The H·G_2_ solutions were concentrated under a nitrogen flux and dried under vacuum. The complexes were then dissolved in CDCl_3_ (1.0 mL) with the help of the sonicator (5 to 10 min). The H·G_2_ complex concentration, at the equilibrium–[H.G_2_]_eq_–was evaluated by integration of the ^1^H NMR complex signals (2.5–6.0 range) versus the total integration of the free host plus complexed molecules at 298 K. With the addition of 2 equivalents of guest, the equilibrium (1) is established:

(1) $$H\;+\;2\;G\;⇄\;H\cdot G_{2}$$

(2) $${\left[H\right]}_{\mathrm{eq}}={\left[H\right]}_{i}-{\left[H\cdot G_{2}\right]}_{\mathrm{eq}}$$

(3) $${\left[G\right]}_{\mathrm{eq}}={\left[G\right]}_{i}-2{\left[H\cdot G_{2}\right]}_{\mathrm{eq}}$$

where [G]_i_ = 2[H]_i_. The *K*_aTOT_ (=*K*_a1_*·K*_a2_, where *K*_a1_ is the equilibrium constant associated with the formation of the monometallic complex, *K*_a2_ is the equilibrium constant associated with the formation of the bimetallic complex, starting from the monometallic one), was calculated as follows:

(4) $$K_{a}=\frac{{\left[H\cdot G\right]}_{\mathrm{eq}}}{{\left[H\right]}_{\mathrm{eq}}\times {\left[G\right]}_{\mathrm{eq}}^{2}}$$

for the equilibrium (1).

In order to have the reliable integration values, the delay times (D1) among successive scans, in the ^1^H NMR, were set at 5 s.

[**5**·2Na]^2+^ 2TFPB¯: white amorphous solid. ^1^H NMR (400 MHz, CDCl_3_) δ: 7.68 (16H, s, TFPB-*o*-*H*), 7.52 (8H, s, TFPB-*p*-*H*), 4.82 (4H, d, *J* 16.5 Hz, O=C-C*H*H-N-*i*Bu), 4.59 (4H, d, *J* 16.6 Hz, O=C-C*H*H-N-*i*Pr), 3.96 (4H, ept, *J* 6.8 Hz, -C*H*(CH_3_)_2_), 3.72 (4H, d, *J* 16.6 Hz, O=C-CH*H*-N-*i*Pr), 3.63 (4H, d, *J* 16.5 Hz, O=C-CH*H*-N-*i*Bu), 3.14 (4H, dd, *J* 14.7, 7.2 Hz, N-C*H*H-CH(CH_3_)_2_), 2.92 (4H, dd, *J* 14.7, 7.2 Hz, N-CH*H*-CH(CH_3_)_2_), 1.83 (4H, m, N-CH_2_-C*H*(CH_3_)_2_), 1.18 (12H, d, *J* 5.6 Hz, N-CH(C*H_3_*)(CH_3_)), 1.03 (12H, d, *J* 5.6 Hz, N-CH(CH_3_)(C*H*_3_)), 0.95 (24H, d, *J* 5.3 Hz, N-CH_2_-CH(C*H_3_*)*_2_*); ^13^C-NMR (100 MHz, CDCl_3_, the TFPB numbering is reported in the S.I.) δ: 170.0 (x4), 168.1 (x4), 161.7 (q, *J* 50 Hz, C-1), 136.2, 134.8, (C-2), 128.8 (q, *J* 30 Hz, C-3), 124.6 (q, *J* 270 Hz, C-5), 117.4 (C-4), 56.1 (x6), 48.4 (x2), 48.1 (x4), 42.2 (x4), 27.3 (x4), 20.9 (x4), 19.9 (x12).

[**6**·2Na]^2+^ 2TFPB¯: white amorphous solid. ^1^H NMR (400 MHz, CDCl_3_) δ: 7.69 (16H, s, TFPB-*o*-*H*), 7.52 (8H, s, TFPB-*p*-*H*), 7.43-7.41 (6H, overlapping, Ar-*H)*, 7.13 (4H, d, *J* 6.9 Hz, Ar-*H)*, 4.97 (2H, d, *J* 17.0 Hz, O=C-C*H*H-N), 4.88 (2H, d, *J* 16.8 Hz, O=C-C*H*H-N-CH_3_), 4.86 (2H, d, *J* 16.7 Hz, O=C-C*H*H-N), 4.72 (2H, d, *J* 16.7 Hz, O=C-C*H*H-N), 4.70 (2H, d, *J* 16.7 Hz, O=C-CH_2_-N-C*H*H-Ph), 4.22 (2H, d, *J* 16.7 Hz, O=C-CH_2_-N-CH*H*-Ph), 3.66 (2H, d, *J* 17.0 Hz, O=C-CH*H*-N), 3.65 (2H, d, *J* 16.7 Hz, O=C-CH*H*-N), 3.54 (2H, d, *J* 16.8 Hz, O=C-CH*H*-N-CH_3_), 3.53 (2H, d, *J* 16.7 Hz, O=C-C*H*H-N), 3.10 (6H, overlapping, N-C*H_2_*-CH(CH_3_)_2_), 2.85 (6H, s, N-C*H_3_*), 2.80 (2H, overlapping, N-C*H*_2_-CH(CH_3_)_2_), 2.01 (4H, m, N-CH_2_-C*H*(CH_3_)_2_), 0.85 (24H, d, *J* 6.3 Hz, N-CH_2_-CH(C*H*_3_)_2_); ^13^C-NMR (150 MHz, CDCl_3_, the TFPB numbering is reported in the S.I.) δ: 169.2 (x4), 161.7 (q, *J* 50 Hz, C-1), 161.5 (x4), 136.2, 134.8, C-2), 129.7 (x2), 129.0 (x2), 128.8 (x4), 128.8 (q, *J* 30 Hz, C-3), 126.5 (x4), 124.6 (q, *J* 270 Hz, C-5), 117.4 (C-4), 34.1 (x4), 30.1 (x4), 29.7 (x4), 27.3 (x3), 22.6 (x3), 22.3 (x2), 19.7 (x4), 14.0 (x4).

[**7**·2Na]^2+^ 2TFPB¯: white amorphous solid. ^1^H NMR (400 MHz, CDCl_3_) δ: 7.71 (16H, s, TFPB-*o*-*H*), 7.53 (8H, s, TFPB-*p*-*H*), 7.38 (12H, bs, Ar-*m*-*H* and Ar-*p*-*H*), 7.11 (8H, d, *J* 6.8 Hz, Ar-*o*-*H*), 4.95 (4H, d, *J* 16.7 Hz, O=C-C*H*H-N-*i*Bu), 4.86 (4H, d, *J* 16.5 Hz, O=C-C*H*H-N-Bn), 4.71 (4H, d, *J* 16.6 Hz, N-C*H*H-Ph), 4.20 (4H, d, *J* 16.6 Hz, N-CH*H*-Ph), 3.62 (4H, d, *J* 16.5 Hz, O=C-CH*H*-N-Bn), 3.60 (4H, d, *J* 16.7 Hz, O=C-N-CH*H*-N-*i*Bu), 2.97 (4H, dd, *J* 15.2 and 7.7 Hz, N-C*H*H-CH(CH_3_)_2_), 2.77 (4H, dd, *J* 15.2 and 7.7 Hz, -CH*H*-CH(CH_3_)_2_), 1.50 (4H, m, N-CH_2_-C*H*(CH_3_)_2_), 0.77 (12H, d, *J* 6.6 Hz, N-CH_2_-CH(C*H*_3_)(CH_3_)), 0.65 (12H, d, *J* 6.6 Hz, -CH_2_-CH(CH_3_)(C*H*_3_)). ^13^C NMR: (100 MHz, CDCl_3_, the TFPB numbering is reported in the S.I.) δ: 169.9 (x4), 169.3 (x4), 161.7 (q, *J* 50 Hz, C-1), 134.8 (C-2), 133.2 (x6), 129.6 (x6), 128.9 (q, *J* 30 Hz C-3), 126.5 (x6), 127.3 (x6), 124.7 (q, *J* 270 Hz, C-5), 117.5 (C-4), 55.9 (x4), 52.0 (x4), 48.1 (x8), 27.2 (x4), 19.7 (x4), 19.4 (x4).

[**8**·2Na]^2+^ 2TFPB¯: white amorphous solid. ^1^H NMR (400 MHz, CDCl_3_) δ: 7.68 (16H, s, TFPB-*o*-*H*), 7.53 (8H, s, TFPB-*p*-*H*), 4.88 (4H, d, *J* 16.8 Hz, O=C-C*H*H-N-CH_3_), 4.70 (4H, d, *J* 17.0 Hz, O=C-C*H*H-N-CH_2_CH_2_CH_2_CH_2_CH_3_), 3.58 (4H, d, *J* 17.0 Hz, O=C-CH*H*-N-CH_2_CH_2_CH_2_CH_2_CH_3_), 3.51 (4H, d, *J* 16.8 Hz, O=C-CH*H*-N-CH_3_), 3.26 (4H, m, O=C-CH_2_-N-C*H*HCH_2_CH_2_CH_2_CH_3_), 3.03 (4H, m, O=C-CH_2_-N-CH*H*CH_2_CH_2_CH_2_CH_3_), 2.88 (12H, s, O=C-CH_2_-N-C*H_3_*), 1.59–1.48 (8H, m, overlapping signals, O=C-CH_2_-N-CH_2_C*H_2_*CH_2_CH_2_CH_3_), 1.26 (16H, m, O=C-CH_2_-N-CH_2_CH_2_C*H_2_*C*H_2_*CH_3_), 0.87 (12H, m, O=C-CH_2_-N-CH_2_CH_2_CH_2_CH_2_C*H_3_*); ^13^C-NMR (100 MHz, CDCl_3_, the TFPB numbering is reported in the S.I.) δ: 170.3 (x4), 168.8 (x4), 160.1 (q, *J* 50 Hz, C-1), 136.4, 134.8 (C-2), 129.0 (q, *J* 30 Hz, C-3), 124.6 (q, *J* 270 Hz, C-5), 117.6 (C-4), 50.2 (x4), 48.8 (x4), 45.5 (x4), 35.8 (x4), 29.7 (x4), 22.2 (x8), 13.6 (x4).

#### 3.1.5. ^1^H NMR Variable Temperature Experiments

The cyclopeptoid was dissolved in C_2_D_2_Cl_4_ (TCDE, 5.0 mM solution), then ^1^H NMR spectra were acquired at different temperatures, increasing 10 Kelvin each time. For compound **5** coalescence was observed at 383 K ($\Delta G_{c}^{\neq }$= 17.4 ± 0.9 kcal/mol) for the signals at 3.66 ppm and 2.32 ppm. For compound **6** coalescence was observed at 363 K ($\Delta G_{c}^{\neq }$= 15.9 ± 0.8 kcal/mol) for the signals at 4.57 ppm and 3.11 ppm. For compound **7** coalescence was observed at 363 K ($\Delta G_{c}^{\neq }$= 16.4 ± 0.8 kcal/mol) for the signals at 5.25 ppm and 3.72 ppm. The $\Delta G_{c}^{\neq }$ was evaluated according to the following relation:

(5) $$\Delta G_{c}^{\neq }=aT_{c}\left[9.972+\log\left(\frac{T_{c}}{\sqrt{\Delta \nu^{2}+6J^{2}}}\right)\right]\;$$

where $T_{c}$ is the coalescence temperature, Δν is the difference in Hertz between the two coupled signals and *J* is the coupling constant between the two signals [42].

For compounds [5–8·2Na]^2+^·2[TFPB]^2^¯ no coalescence was observed up to 383 K.

#### 3.1.6. General Procedure for the Pirkle’s Alcohol Addition to Racemic Mixture of Cyclopeptoid 7

To a 5.0 mM solution of cyclic peptoid **7** in CDCl_3_ (0.5 mL), 1.0 equivalent of Pirkle’s alcohol ((*R*)-1-(9-anthryl)-2,2,2-trifluoroethanol) was added. After the addition, the mixture was mixed for 1 min and the ^1^H NMR spectrum was recorded. Further 1.0 equivalent of Pirkle’s alcohol was added in order to increase the protons resonances’ splitting. NMR spectra were recorded on a Bruker DRX 600 (^1^H at 600.13 MHz).

### 3.2. Assay of Insecticidal Activity against Silkworm Larvae

Third-instar larvae of silkworm *Bombyx mori* were purchased from Kougensha Co, Ltd. (Matsumoto, Japan) and cultured on an artificial diet SilkMate 2S obtained from Nosan Corporation. Each compound dissolved or suspended in DMSO was injected into open vessels of 4th-instar larvae (mean body weight *ca* 0.9 g) resulting in the dose of 300 nmol/larva. A total of 10 larvae were tested and the number of dead larvae was counted.

### 3.3. Cell Lines and Viability Assay

A375 (human melanoma) cells (American Type Culture Collection, Manassas, VA, USA) were maintained in DMEM supplemented with 10% heat-inactivated foetal bovine serum (Invitrogen (Carslbad, CA, USA) in 5% CO_2_ humid atmosphere. To ensure logarithmic growth, cells were subcultured every 2 days. The number of viable cells was determined by MTT conversion assay using [3-4,5-dimethyldiazol-2-yl]-2,5-diphenyl tetrazolium bromide (MTT, Sigma-Aldrich, Saint Louis, MO, USA). Briefly, A375 or A549 (3000/well) cells were seeded in triplicate in 96 well/plates and incubated with increasing concentrations of compounds **5**–**8** (between 5 µM and 50 µM) or DMSO 0.10% (*v*/*v*) for 72 h in DMEM medium with 10% FBS. Following the treatment, 20 µL of MTT (5 mg/mL in PBS) was added and the cells were incubated for additional 3 h at 37 °C. The formazan crystals thus formed were dissolved in 100 μL of buffer containing 50% (*v*/*v*) *N*,*N*-dimethylformamide, 20% SDS (pH 4.5). The absorbance was measured at 570 nm with a Multiskan™ GO Microplate Spectrophotometer (Thermo Fisher Scientific, Waltham, MA, USA).

## 4. Conclusions

Understanding the conformational features of cyclooligoamides represents a necessary step to exploit their biological properties. In particular, the comprehension of their stereochemical traits is crucial, especially when the generation of rigid structures induces the formation of conformational enantiomorphs.

The isosteric transformation of bioactive cyclooctadepsipeptides in cyclooctapeptoids represents a simple way to explore the molecular space conjunct to macrocyclic oligoamides. Combined NMR and computational techniques allowed us to assign the structural identity of the formed species. The intrinsic conformational stability of cyclooctamer peptoids, in their free and complexed form, clarified their intricate topological features and evidenced possible detrimental effects on the binding interactions with target biomolecules and their scarce biological activities on silkworm larvae.

Differently from conformationally heterogeneous cyclohexapeptoids [10,12], cyclic octamer peptoids show limited capacity to mimic the natural counterparts. Although they are perfectly suited for solid-phase synthesis and combinatorial approaches to drug discovery, they show undesirable conformational and stereochemical complication that can decrease their adaptability to the molecular target.

Additional efforts are currently underway in order to design libraries of diverse and more malleable compounds that can exhibit increased biological properties and higher conformational mobility.

## Figures and Tables

**Table 1:** Solid-phase synthesis of linear peptoids **9**–**12**: sequences, mass data (ESI/MS), chemical yields and purity.

Sequence ^a^ (Oligomer)	ESI/MS	Yield	Purity
H-[*N*Val-*N*Leu]_4_-OH (**9**)	867.5 [M + H]^+^	100%	82%
H-[*N*Phe-*N*Leu-*N*Ala-*N*Leu]_2_-OH (**10**)	908.2 [M + H]^+^	49%	92%
H-[*N*Phe-*N*Leu]_4_-OH (**11**)	1059.6 [M + H]^+^	89%	71%
H-[*N*am-*N*Ala]_4_-OH (**12**)	811.5 [M + H]^+^	100%	83%

^a^*N*Val = *N*-(isopropyl)glycine; *N*Leu = *N*-(isobutyl)glycine; *N*Phe = *N*-(benzyl)glycine; *N*Ala = *N*-(methyl)glycine; *N*am = *N*-(pentyl)glycine.

**Table 2:** Toxicity of **5**–**8** against silkworms by haemolymph injection ^a^.

Compound	Number of Dead Larvae (*n* = 10)					
	Time after Injection (h)					
	1	6	24	72	120	168
vehicle	0	0	0	0	0	0
**5**	0	0	0	2	2	2
**6**	0	0	0	1	2	2
**7**	0	0	0	2	2	2
**8**	0	0	0	1	1	1

^a^ Each compound in DMSO was injected into 4th-instar larvae (*ca* 0.9 g body weight) at a dose of 300 nmol/larva.

## Supplementary Materials

The following are available online.
