# Supplementary material for: Cyclic Octamer Peptoids: Simplified Isosters of Bioactive Fungal Cyclodepsipeptides
Source: Molecules. 2018 Jul 19;23(7):1779. doi: 10.3390/molecules23071779 (PMC6100324; doi:10.3390/molecules23071779)
Supplement: Supplementary file 1 [file molecules-23-01779-s001.zip › SUPPORTING MATERIAL De Riccardis.doc]

# *Supplementary Material*

# *for*

Cyclic Octamer Peptoids: Simplified Isosters of Bioactive Fungal Cyclodepsipeptides

Assunta D’Amato ^1^, Giorgio Della Sala ^1^, Irene Izzo ^1^, Chiara Costabile ^1^, Yuichi Masuda, ^2,^* and Francesco De Riccardis ^1,^*

1 Department of Chemistry and Biology “A. Zambelli”, University of Salerno, Via Giovanni Paolo II, 132, Fisciano (SA), 84084, Italy
2 Graduate School of Bioresources, Mie University, 1577 Kurimamachiya-cho, Tsu, 514-8507, Japan

*****	Correspondence: dericca@unisa.it; Tel.: +39-089-969-552; masuda@bio.mie-u.ac.jp; Tel.: +81-59-231-9620

**0.5 equiv.**

**1.5 equiv.**

**1.0 equiv.**

**2.0 equiv.**

Figure S1. TFPB anion numbering as reported in the ^13^C NMR assignment of the [5-8^.^2Na]^2+^ complexes.

Figure S2. ^1^H NMR assignment for compounds 5a, 7b, and 6.

1.0 Computational details

**1.1 Minimum energy structures of 5a, 5b,** and **7a, 7b**

The DFT calculations were performed with the Gaussian09 set of programs,

^[1]^ using the BP86 functional of Becke and Perdew.^[2]^ The electronic configuration of the molecular systems was described with the standard triple zeta valence basis set with a polarization function of Ahlrichs and co-workers for H, C, N, O (TZVP keyword in Gaussian).^[3]^ The geometry optimizations were performed without symmetry constraints, and the characterization of the located stationary points was performed by analytical frequency calculations. Solvent effects including contributions of non electrostatic terms have been estimated in single-point calculations on the gas phase optimized structures, based on the polarizable continuous solvation model PCM using CHCl_3_ as a solvent.^[4]^

**Figure S3.** Top and side view of minimum energy structures of conformational diastereisomers **5a/b** and **7a/b** with the relative *cis*/*trans* (**c**/**t**) amide bond reported. Isobutyl groups are highlighted in green, whereas isopropyl and benzyl groups in yellow.

**1.2 Cartesian coordinates and energies of calculated structures**

140

**5a** E(gas)=-2765.71509690 G(gas)=-2764.634749 E(CHCl3)=-2765.73961904

O	0.590100	3.912748	0.943849

O	4.550320	3.278578	-0.216324

O	3.616608	-0.183172	-1.879793

O	1.466974	-1.396499	0.208776

N	2.426679	2.624845	1.416353

N	5.422191	1.155205	-0.289546

N	3.818301	-2.195138	-0.824762

N	0.134233	-2.484881	-1.292349

C	1.322968	2.961459	0.665562

C	3.348951	1.572816	1.026626

H	2.807103	0.731031	0.571827

H	3.806668	1.166896	1.944871

C	2.838566	3.503547	2.520554

H	2.207956	4.401763	2.454997

H	3.882776	3.809150	2.339553

C	2.695418	2.870056	3.919720

C	4.482640	2.088004	0.096957

C	5.220080	-0.264190	-0.059988

H	4.922293	-0.441166	0.985031

H	6.180469	-0.783555	-0.194323

C	6.579329	1.600415	-1.107880

C	6.635663	0.909083	-2.475431

H	6.386377	2.671482	-1.262387

C	7.889427	1.445489	-0.321282

C	4.149127	-0.874682	-1.006679

C	2.617285	-2.644510	-1.518924

H	2.619311	-3.743107	-1.547662

H	2.641864	-2.267084	-2.550220

C	4.274653	-3.040148	0.289677

H	3.397656	-3.283702	0.914043

H	4.961486	-2.463206	0.923189

C	4.991428	-4.334634	-0.150916

C	1.348863	-2.121859	-0.788444

C	1.040120	2.045295	-0.548471

H	1.906464	2.019093	-1.224170

H	0.870985	1.016587	-0.194947

C	-0.065239	-3.304078	-2.514596

H	0.927395	-3.675801	-2.802074

C	-0.955024	-4.526511	-2.247155

C	-0.595272	-2.441414	-3.672526

O	-0.590162	-3.912745	0.944051

O	-4.550295	-3.278616	-0.216132

O	-3.616660	0.183120	-1.879960

O	-1.467036	1.396435	0.208689

N	-2.426648	-2.624689	1.416491

N	-5.422144	-1.155245	-0.289610

N	-3.818316	2.195099	-0.824938

N	-0.134224	2.484717	-1.292447

C	-1.322992	-2.961459	0.665673

C	-3.348930	-1.572719	1.026624

H	-2.807087	-0.730981	0.571727

H	-3.806637	-1.166668	1.944819

C	-2.838583	-3.503298	2.520757

H	-2.207987	-4.401532	2.455296

H	-3.882793	-3.808893	2.339749

C	-2.695462	-2.869663	3.919865

C	-4.482609	-2.088006	0.097019

C	-5.220092	0.264168	-0.060119

H	-4.922272	0.441207	0.984880

H	-6.180507	0.783489	-0.194446

C	-6.579299	-1.600552	-1.107866

C	-6.635870	-0.909122	-2.475355

H	-6.386220	-2.671583	-1.262472

C	-7.889337	-1.445867	-0.321115

C	-4.149184	0.874652	-1.006868

C	-2.617268	2.644435	-1.519070

H	-2.619273	3.743031	-1.547823

H	-2.641826	2.266999	-2.550364

C	-4.274647	3.040104	0.289513

H	-3.397651	3.283618	0.913895

H	-4.961512	2.463174	0.923003

C	-4.991372	4.334622	-0.151071

C	-1.348877	2.121751	-0.788557

C	-1.040135	-2.045413	-0.548442

H	-1.906474	-2.019355	-1.224158

H	-0.871066	-1.016650	-0.195055

C	0.065300	3.303904	-2.514696

H	-0.927319	3.675638	-2.802209

C	0.955112	4.526312	-2.247257

C	0.595340	2.441203	-3.672598

C	1.235872	2.524936	4.244562

H	3.283710	1.932132	3.940096

C	3.297667	3.817822	4.969083

H	1.158204	2.026396	5.223631

H	0.619027	3.437429	4.275498

H	0.801060	1.856755	3.487231

H	3.237492	3.380126	5.977335

H	4.356266	4.039216	4.758995

H	2.751910	4.775771	4.988694

C	5.270617	-5.205737	1.083660

H	4.306741	-4.897910	-0.811001

C	6.275929	-4.038125	-0.935759

H	5.755589	-6.150654	0.795989

H	5.944565	-4.688398	1.787247

H	4.343819	-5.452380	1.624037

H	6.739865	-4.966724	-1.301531

H	6.076930	-3.395980	-1.806901

H	7.017083	-3.528947	-0.296046

C	-3.297757	-3.817285	4.969330

C	-1.235917	-2.524520	4.244687

H	-3.283735	-1.931726	3.940102

H	-1.158252	-2.025793	5.223661

H	-0.619088	-3.437018	4.275826

H	-0.801067	-1.856494	3.487239

H	-3.237590	-3.379463	5.977526

H	-4.356358	-4.038667	4.759237

H	-2.752027	-4.775246	4.989080

C	-5.270530	5.205727	1.083512

H	-4.306661	4.897879	-0.811148

C	-6.275882	4.038169	-0.935920

H	-5.755457	6.150668	0.795846

H	-5.944506	4.688413	1.787090

H	-4.343723	5.452321	1.623896

H	-6.739782	4.966789	-1.301684

H	-6.076900	3.396026	-1.807067

H	-7.017056	3.529009	-0.296215

H	0.704582	3.055194	-4.579753

H	1.581579	2.013443	-3.437377

H	-0.093288	1.612863	-3.894684

H	0.984841	5.160029	-3.146835

H	0.570993	5.114570	-1.403598

H	1.990104	4.235309	-2.012804

H	8.729925	1.852896	-0.903679

H	7.840976	1.985806	0.635186

H	8.121115	0.387995	-0.110713

H	7.433741	1.363030	-3.082479

H	6.865712	-0.165529	-2.386459

H	5.681075	1.011133	-3.007103

H	-0.704484	-3.055429	-4.579667

H	-1.581523	-2.013667	-3.437334

H	0.093345	-1.613066	-3.894617

H	-0.984703	-5.160262	-3.146711

H	-0.570917	-5.114726	-1.403460

H	-1.990034	-4.235537	-2.012746

H	-8.729854	-1.853300	-0.903468

H	-7.840722	-1.986291	0.635284

H	-8.121113	-0.388423	-0.110394

H	-7.433924	-1.363162	-3.082365

H	-6.866106	0.165441	-2.386283

H	-5.681311	-1.010968	-3.007119

140

**5b** E(gas)=-2765.71273317 G(gas)=-2764.631156 E(CHCl3)=-2765.73729007

O	-1.508104	-3.675019	1.591504

O	-4.848546	-2.190417	-0.314810

O	-3.340515	1.280476	-1.436180

O	-1.165514	1.588858	0.807323

N	-3.103387	-2.046172	1.852925

N	-5.442631	0.023679	-0.228656

N	-3.294922	3.082623	-0.042814

N	0.352907	2.568845	-0.597130

C	-2.018901	-2.612215	1.224128

C	-3.734147	-0.830446	1.368411

H	-2.973925	-0.089037	1.069991

H	-4.278854	-0.376157	2.210963

C	-3.679408	-2.737575	3.033031

C	-3.417620	-1.939520	4.319257

H	-3.108154	-3.675301	3.083853

C	-5.163407	-3.073691	2.844596

C	-4.719113	-1.075466	0.192152

C	-5.112948	1.370639	0.210614

H	-4.978424	1.392567	1.301318

H	-5.973884	2.022343	-0.006962

C	-6.274550	-0.091725	-1.438280

H	-5.886753	0.618635	-2.187696

H	-6.117196	-1.107765	-1.827183

C	-7.777479	0.146922	-1.191894

C	-3.840331	1.911587	-0.500165

C	-1.980403	3.410453	-0.579152

H	-1.721358	4.434177	-0.274332

H	-2.014292	3.382482	-1.677189

C	-3.851544	3.914336	1.051875

C	-2.945000	3.928707	2.291951

H	-4.805227	3.447532	1.332690

C	-4.175919	5.333590	0.555020

C	-0.895426	2.433181	-0.055285

C	-1.438218	-1.822083	0.027502

H	-2.215601	-1.625775	-0.722768

H	-1.051372	-0.852837	0.377319

C	0.722901	3.639706	-1.538233

H	0.150373	4.549959	-1.302964

H	1.775965	3.893056	-1.338209

C	0.554336	3.287382	-3.032859

O	1.508245	3.675297	1.591419

O	4.848691	2.190331	-0.315046

O	3.340671	-1.280574	-1.436254

O	1.165428	-1.588661	0.807286

N	3.103537	2.046439	1.852700

N	5.442719	-0.023760	-0.228585

N	3.294790	-3.082575	-0.042724

N	-0.352998	-2.568641	-0.597173

C	2.018955	2.612445	1.224056

C	3.734133	0.830609	1.368262

H	2.973826	0.089285	1.069821

H	4.278673	0.376231	2.210865

C	3.679710	2.737849	3.032717

H	3.108803	3.675798	3.083368

C	5.163856	3.073344	2.844361

C	3.417505	1.940058	4.319028

C	4.719138	1.075449	0.191998

C	5.112907	-1.370670	0.210757

H	4.978307	-1.392508	1.301451

H	5.973800	-2.022470	-0.006704

C	6.274697	0.091460	-1.438212

H	5.886692	-0.618767	-2.187637

H	6.117629	1.107566	-1.827069

C	7.777573	-0.147571	-1.191827

C	3.840294	-1.911567	-0.500053

C	1.980279	-3.410313	-0.579140

H	1.721148	-4.434024	-0.274352

H	2.014226	-3.382331	-1.677176

C	3.851226	-3.914267	1.052085

C	2.944642	-3.928346	2.292133

H	4.804990	-3.447618	1.332875

C	4.175359	-5.333636	0.555402

C	0.895346	-2.432967	-0.055339

C	1.438125	1.822267	0.027525

H	2.215441	1.625847	-0.722786

H	1.051237	0.853073	0.377439

C	-0.722983	-3.639550	-1.538225

H	-0.150461	-4.549789	-1.302890

H	-1.776058	-3.892887	-1.338236

C	-0.554347	-3.287305	-3.032862

C	-0.834191	-4.539521	-3.878848

C	-1.448585	-2.115595	-3.460153

H	0.497278	-2.987958	-3.194284

H	-0.691197	-4.329634	-4.949632

H	-0.167893	-5.374234	-3.607456

H	-1.873678	-4.881634	-3.743010

H	-1.301347	-1.884174	-4.526564

H	-2.513998	-2.358523	-3.313676

H	-1.227595	-1.202305	-2.889848

H	-5.513048	-3.683244	3.691951

H	-5.791484	-2.168037	2.813629

H	-5.324484	-3.632085	1.913350

H	-3.765942	-2.509907	5.194131

H	-2.344099	-1.737586	4.442517

H	-3.953613	-0.975688	4.324271

C	-8.516040	0.164365	-2.539770

C	-8.373151	-0.899093	-0.240179

H	-7.902120	1.145246	-0.728709

H	-9.435998	-0.691942	-0.040081

H	-8.298436	-1.908571	-0.675296

H	-7.843269	-0.913463	0.723719

H	-9.589674	0.361790	-2.399374

H	-8.115013	0.937724	-3.213903

H	-8.419965	-0.808313	-3.049796

C	0.834323	4.539526	-3.878904

C	1.448509	2.115578	-3.460029

H	-0.497299	2.988095	-3.194333

H	0.691372	4.329588	-4.949684

H	0.168075	5.374305	-3.607598

H	1.873828	4.881569	-3.743029

H	1.301340	1.884131	-4.526445

H	2.513931	2.358415	-3.313473

H	1.227384	1.202328	-2.889711

C	8.373444	0.898381	-0.240151

H	7.902009	-1.145883	-0.728595

C	8.516144	-0.165169	-2.539683

H	9.436189	0.690936	-0.039799

H	8.299179	1.907800	-0.675497

H	7.843382	0.913117	0.723638

H	9.589754	-0.362687	-2.399237

H	8.115065	-0.938522	-3.213789

H	8.420168	0.807499	-3.049742

H	-4.682113	5.902524	1.349475

H	-3.263765	5.888553	0.287144

H	-4.833958	5.305582	-0.325868

H	-3.439766	4.477639	3.107877

H	-2.718359	2.908345	2.627645

H	-1.985181	4.426658	2.088549

H	3.766128	2.510341	5.193851

H	2.343878	1.738709	4.442297

H	3.953007	0.975949	4.324126

H	5.513643	3.682907	3.691655

H	5.791597	2.167449	2.813652

H	5.325254	3.631502	1.913034

H	4.681489	-5.902547	1.349915

H	3.263113	-5.888497	0.287626

H	4.833379	-5.305846	-0.325508

H	3.439251	-4.477355	3.108105

H	2.718237	-2.907914	2.627772

H	1.984709	-4.426071	2.088723

156

**7a** E(gas)=-3375.58445873 G(gas)=-3374.426190 E(CHCl3)=-3375.61287099

O	1.120868	-4.087440	-1.634027

O	4.460554	-2.856223	0.375546

O	3.591767	0.993173	1.323891

O	1.483127	1.542064	-1.071355

N	2.834500	-2.581019	-1.839283

N	5.374534	-0.758554	0.208734

N	3.837165	2.620718	-0.250998

N	0.172981	2.771236	0.344063

C	1.664712	-3.038456	-1.279265

C	3.544610	-1.413591	-1.348828

H	2.843957	-0.616977	-1.051433

H	4.120917	-0.997991	-2.190148

C	3.555931	-3.442529	-2.787488

H	3.032805	-4.409468	-2.790967

H	4.572881	-3.609472	-2.391656

C	3.625205	-2.884561	-4.223610

C	4.494773	-1.751613	-0.168887

C	5.306880	0.589554	-0.332380

H	5.179254	0.546831	-1.421936

H	6.278882	1.078111	-0.155737

C	6.174449	-0.946674	1.432144

H	5.789958	-0.270791	2.213322

H	5.981862	-1.979852	1.755719

C	7.655655	-0.717043	1.208001

C	4.170836	1.417450	0.318445

C	2.614196	3.227727	0.259270

H	2.552414	4.259664	-0.116428

H	2.656271	3.261467	1.355868

C	4.360000	3.149249	-1.518674

H	3.522421	3.211156	-2.234709

H	5.081888	2.434528	-1.936114

C	5.057173	4.521880	-1.393508

C	1.369265	2.431579	-0.219156

C	1.039553	-2.152175	-0.174155

H	1.749270	-2.012511	0.655633

H	0.782742	-1.163046	-0.580338

C	-0.017205	3.860459	1.309116

H	0.850988	4.533271	1.266112

H	-0.872872	4.463396	0.969243

C	-0.238026	3.401126	2.744028

O	-1.120638	4.087790	-1.633348

O	-4.460654	2.856235	0.375901

O	-3.591611	-0.993387	1.323484

O	-1.483045	-1.541831	-1.071771

N	-2.834393	2.581546	-1.838811

N	-5.374429	0.758504	0.208690

N	-3.837085	-2.620703	-0.251634

N	-0.172916	-2.771208	0.343494

C	-1.664565	3.038786	-1.278741

C	-3.544507	1.413980	-1.348684

H	-2.843867	0.617291	-1.051465

H	-4.120784	0.998603	-2.190137

C	-3.555711	3.443237	-2.786927

H	-3.032703	4.410238	-2.790007

H	-4.572774	3.609941	-2.391288

C	-3.624538	2.885679	-4.223238

C	-4.494755	1.751721	-0.168723

C	-5.306699	-0.589459	-0.332746

H	-5.178904	-0.546457	-1.422272

H	-6.278723	-1.078071	-0.156367

C	-6.174445	0.946365	1.432086

H	-5.789916	0.270433	2.213202

H	-5.981997	1.979530	1.755781

C	-7.655602	0.716588	1.207809

C	-4.170723	-1.417515	0.318000

C	-2.614096	-3.227797	0.258493

H	-2.552260	-4.259626	-0.117493

H	-2.656181	-3.261850	1.355080

C	-4.359938	-3.148965	-1.519418

H	-3.522409	-3.210587	-2.235540

H	-5.081953	-2.434220	-1.936596

C	-5.056960	-4.521700	-1.394560

C	-1.369189	-2.431477	-0.219708

C	-1.039478	2.152319	-0.173738

H	-1.749232	2.012511	0.655996

H	-0.782663	1.163260	-0.580092

C	0.017225	-3.860573	1.308388

H	-0.850760	-4.533633	1.264935

H	0.873160	-4.463210	0.968661

C	0.237492	-3.401540	2.743485

C	4.514933	-3.799164	-5.080431

H	4.106421	-1.887759	-4.189091

C	2.229204	-2.719145	-4.839462

H	4.614995	-3.406057	-6.103636

H	5.526915	-3.898172	-4.656229

H	4.080809	-4.810029	-5.152950

H	2.293728	-2.279291	-5.847045

H	1.723733	-3.694418	-4.924037

H	1.592168	-2.066805	-4.224588

C	5.420894	5.035006	-2.795277

H	4.335286	5.233074	-0.951996

C	6.286789	4.455334	-0.478659

H	5.883920	6.031577	-2.741230

H	6.141983	4.360895	-3.287494

H	4.533551	5.110770	-3.442793

H	6.737675	5.451154	-0.350323

H	6.027158	4.072541	0.519676

H	7.059293	3.794989	-0.907863

C	-4.514166	3.800391	-5.080047

C	-2.228346	2.720632	-4.838742

H	-4.105625	1.888793	-4.189136

H	-2.292506	2.281100	-5.846486

H	-1.722938	3.695982	-4.922846

H	-1.591403	2.068174	-4.223890

H	-4.613977	3.407510	-6.103363

H	-5.526241	3.899193	-4.656015

H	-4.080132	4.811318	-5.152254

C	-5.420748	-5.034492	-2.796433

H	-4.334973	-5.232945	-0.953296

C	-6.286508	-4.455503	-0.479592

H	-5.883710	-6.031106	-2.742602

H	-6.141914	-4.360303	-3.288430

H	-4.533446	-5.110042	-3.444031

H	-6.737282	-5.451399	-0.351459

H	-6.026830	-4.072921	0.518811

H	-7.059111	-3.795130	-0.908573

C	-1.171107	4.074519	3.547448

C	-1.342493	3.722495	4.890304

C	-0.583305	2.686079	5.443771

C	0.341475	2.002139	4.645826

C	0.513047	2.354818	3.303131

H	-1.774790	4.876682	3.113677

H	-2.077265	4.251976	5.501218

H	-0.716411	2.408001	6.491852

H	0.933287	1.186229	5.066304

H	1.231203	1.804359	2.689274

C	8.359198	0.221927	1.977444

C	9.734051	0.416085	1.792671

C	10.422163	-0.326441	0.828826

C	9.728913	-1.265126	0.052919

C	8.358344	-1.458530	0.241839

H	7.824350	0.803269	2.734099

H	10.265704	1.150589	2.402028

H	11.494095	-0.177137	0.681832

H	10.262053	-1.853571	-0.697561

H	7.821949	-2.196495	-0.360317

C	1.170122	-4.075273	3.547150

C	1.341018	-3.723569	4.890151

C	0.581790	-2.687131	5.443525

C	-0.342538	-2.002861	4.645345

C	-0.513624	-2.355219	3.302499

H	1.773842	-4.877451	3.113456

H	2.075441	-4.253315	5.501254

H	0.714517	-2.409301	6.491720

H	-0.934378	-1.186934	5.065751

H	-1.231440	-1.804498	2.688481

C	-8.359045	-0.222783	1.976852

C	-9.733858	-0.417087	1.791920

C	-10.422026	0.325692	0.828312

C	-9.728876	1.264782	0.052802

C	-8.358353	1.458334	0.241885

H	-7.824154	-0.804318	2.733328

H	-10.265433	-1.151900	2.400973

H	-11.493925	0.176275	0.681194

H	-10.262064	1.853428	-0.697486

H	-7.822037	2.196616	-0.359954

156

**7b** E(gas)=-3375.58567758 G(gas)=-3374.428085 E(CHCl3)=-3375.61343210

O	0.337469	4.091349	0.707454

O	4.272251	3.612150	-0.657516

O	3.762052	-0.046489	-2.087972

O	1.586340	-1.186706	0.035973

N	2.287681	2.967739	1.140640

N	5.326995	1.576450	-0.543056

N	4.033006	-1.929230	-0.827122

N	0.406255	-2.530813	-1.391473

C	1.121246	3.176399	0.441607

C	3.256789	1.945591	0.786581

H	2.748934	1.053660	0.390020

H	3.751550	1.623782	1.718886

C	2.703500	3.960238	2.141316

H	1.900142	4.711848	2.164979

H	3.620683	4.458380	1.786298

C	2.922059	3.369439	3.521561

C	4.321155	2.462809	-0.216531

C	5.246840	0.170041	-0.185962

H	4.923642	0.061434	0.859119

H	6.258992	-0.261588	-0.239837

C	6.301532	1.948360	-1.581152

H	6.238998	1.202603	-2.391522

H	5.970601	2.914460	-1.988587

C	7.749773	2.066926	-1.065458

C	4.279818	-0.609090	-1.120811

C	2.890948	-2.529535	-1.504057

H	2.972267	-3.622183	-1.411847

H	2.924840	-2.268023	-2.569709

C	4.466829	-2.600049	0.411377

H	3.633229	-2.601891	1.135079

H	5.283840	-2.016735	0.854973

C	4.951382	-4.019838	0.171088

C	1.566508	-2.022972	-0.878572

C	0.833158	2.181864	-0.707662

H	1.659549	2.188424	-1.432602

H	0.741987	1.159624	-0.311070

C	0.352910	-3.612259	-2.391182

H	1.190861	-4.305552	-2.223648

H	-0.558519	-4.194087	-2.182861

C	0.344441	-3.152149	-3.865766

O	-0.337775	-4.091520	0.707082

O	-4.272666	-3.612104	-0.657693

O	-3.762304	0.046478	-2.087971

O	-1.586715	1.186641	0.036013

N	-2.287949	-2.967891	1.140365

N	-5.327317	-1.576368	-0.543031

N	-4.033301	1.929304	-0.827228

N	-0.406482	2.530797	-1.391263

C	-1.121530	-3.176535	0.441300

C	-3.257033	-1.945674	0.786428

H	-2.749164	-1.053756	0.389857

H	-3.751698	-1.623872	1.718785

C	-2.703752	-3.960420	2.141005

H	-3.621043	-4.458415	1.786058

H	-1.900486	-4.712137	2.164493

C	-2.922061	-3.369759	3.521353

C	-4.321497	-2.462789	-0.216632

C	-5.247052	-0.169974	-0.185917

H	-4.923709	-0.061400	0.859121

H	-6.259189	0.261707	-0.239651

C	-6.301904	-1.948179	-1.581110

H	-6.239493	-1.202313	-2.391391

H	-5.970960	-2.914196	-1.988719

C	-7.750084	-2.066834	-1.065286

C	-4.280098	0.609134	-1.120860

C	-2.891164	2.529529	-1.504101

H	-2.972460	3.622184	-1.411991

H	-2.924977	2.267916	-2.569731

C	-4.467040	2.600025	0.411376

H	-3.633557	2.601376	1.135214

H	-5.284379	2.016939	0.854671

C	-4.951005	4.020044	0.171300

C	-1.566790	2.022945	-0.878498

C	-0.833443	-2.181961	-0.707937

H	-1.659801	-2.188563	-1.432915

H	-0.742347	-1.159716	-0.311340

C	-0.353012	3.612301	-2.390904

H	-1.190927	4.305641	-2.223373

H	0.558445	4.194051	-2.182495

C	-0.344471	3.152293	-3.865522

C	-0.383822	4.391207	-4.774539

C	0.858937	2.258487	-4.194141

H	-1.263984	2.565941	-4.044599

H	-0.415190	4.098708	-5.834886

H	-1.266027	5.019087	-4.571723

H	0.514018	5.013910	-4.627057

H	0.827095	1.936698	-5.246578

H	1.807833	2.798253	-4.039545

H	0.880200	1.352887	-3.571405

C	8.694559	2.305972	-2.253639

C	7.887983	3.168874	-0.006356

H	8.033915	1.103254	-0.599543

H	8.916757	3.214381	0.383475

H	7.640932	4.153489	-0.434648

H	7.210573	2.995900	0.843023

H	9.742016	2.362172	-1.920385

H	8.621047	1.499898	-3.000879

H	8.454685	3.255653	-2.759660

C	0.384026	-4.390987	-4.774874

C	-0.859042	-2.258458	-4.194432

H	1.263903	-2.565676	-4.044710

H	0.415439	-4.098401	-5.835195

H	1.266299	-5.018763	-4.572033

H	-0.513740	-5.013826	-4.627517

H	-0.827137	-1.936589	-5.246843

H	-1.807888	-2.798348	-4.039966

H	-0.880468	-1.352901	-3.571640

C	-7.888256	-3.169028	-0.006440

H	-8.034091	-1.103264	-0.599067

C	-8.695051	-2.305454	-2.253400

H	-8.916951	-3.214426	0.383619

H	-7.641505	-4.153586	-0.435037

H	-7.210612	-2.996375	0.842815

H	-9.742453	-2.361853	-1.919990

H	-8.621734	-1.499094	-3.000349

H	-8.455228	-3.254920	-2.759853

C	4.072179	3.692767	4.258002

C	4.262360	3.188194	5.550114

C	3.304300	2.343803	6.119668

C	2.156105	2.009630	5.390155

C	1.965771	2.519071	4.102807

H	4.825611	4.349946	3.814042

H	5.163289	3.451433	6.109393

H	3.451132	1.945832	7.126249

H	1.402673	1.351759	5.829530

H	1.069586	2.255290	3.535773

C	4.340513	-5.098816	0.826134

C	4.803682	-6.405807	0.629498

C	5.881607	-6.645669	-0.227558

C	6.493334	-5.574089	-0.891530

C	6.030538	-4.271128	-0.692942

H	3.489163	-4.915490	1.487234

H	4.315306	-7.236640	1.143323

H	6.243288	-7.664455	-0.382826

H	7.334941	-5.756087	-1.563814

H	6.502964	-3.437926	-1.220423

C	-4.071745	-3.693705	4.258206

C	-4.261677	-3.189339	5.550432

C	-3.303812	-2.344528	6.119699

C	-2.156064	-2.009737	5.389773

C	-1.965967	-2.518980	4.102308

H	-4.825039	-4.351201	3.814482

H	-5.162267	-3.453065	6.110029

H	-3.450456	-1.946710	7.126367

H	-1.402783	-1.351531	5.828908

H	-1.070124	-2.254715	3.534960

C	-4.339471	5.098723	0.826221

C	-4.802083	6.405933	0.629726

C	-5.880114	6.646315	-0.227052

C	-6.492510	5.575032	-0.890889

C	-6.030272	4.271853	-0.692441

H	-3.488041	4.914993	1.487105

H	-4.313194	7.236525	1.143452

H	-6.241365	7.665271	-0.382202

H	-7.334208	5.757434	-1.562951

H	-6.503230	3.438877	-1.219803

1. Gaussian 09, Revision A.02, Frisch, M. J.; Trucks, G. W.; Schlegel, H. B.; Scuseria, G. E.; Robb, M. A.; Cheeseman, J. R.; Scalmani, G.; Barone, V.; Mennucci, B.; Petersson, G. A.; Nakatsuji, H.; Caricato, M.; Li, X.; Hratchian, H. P.; Izmaylov, A. F.; Bloino, J.; Zheng, G.; Sonnenberg, J. L.; Hada, M.; Ehara, M.; Toyota, K.; Fukuda, R.; Hasegawa, J.; Ishida, M.; Nakajima, T.; Honda, Y.; Kitao, O.; Nakai, H.; Vreven, T.; Montgomery, J. A., Jr.; Peralta, J. E.; Ogliaro, F.; Bearpark, M.; Heyd, J. J.; Brothers, E.; N. Kudin, K.; Staroverov, V. N.; Kobayashi, R.; Normand, J.; Raghavachari, K.; Rendell, A.; Burant, J. C.; Iyengar, S. S.; Tomasi, J.; Cossi, M.; Rega, N.; Millam, J. M.; Klene, M.; Knox, J. E.; Cross, J. B.; Bakken, V.; Adamo, C.; Jaramillo, J.; Gomperts, R.; Stratmann, R. E.; Yazyev, O.; Austin, A. J.; Cammi, R.; Pomelli, C.; Ochterski, J. W.; Martin, R. L.; Morokuma, K.; Zakrzewski, V. G.; Voth, G. A.; Salvador, P.; Dannenberg, J. J.; Dapprich, S.; Daniels, A. D.; Farkas, O.; Foresman, J. B.; Ortiz, J. V.; Cioslowski, J.; Fox, D. J. Gaussian, Inc., Wallingford CT, **2009**.
2. a) Becke, A. Phys. Rev. A 1988, 38, 3098– 3100. b) Perdew, J. P. Phys. Rev. B 1986, 33, 8822– 8824. c) Perdew, J. P. *Phys. Rev. B* **1986**, *34*, 7406– 7406.
3. Schaefer, A., Horn, H. and Ahlrichs, R. *J. Chem. Phys.* **1994**, *100*, 5829– 5835.
4. a) Barone, V. and Cossi, M. *J. Phys. Chem. A* **1998**, *102*, 1995– 2001. b) Tomasi, J. and Persico, M. *Chem. Rev.* **1994**, *94*, 2027– 2094.
